# Insights from a decade of surveillance: Molecular epidemiology of methicillin-resistant *Staphylococcus aureus* in Norway from 2008 to 2017

**DOI:** 10.1371/journal.pone.0297333

**Published:** 2024-03-12

**Authors:** Torunn Gresdal Rønning, Hege Enger, Jan Egil Afset, Christina Gabrielsen Ås

**Affiliations:** 1 Department of Medical Microbiology, St. Olavs Hospital, Trondheim University Hospital, Trondheim, Norway; 2 Department of Clinical and Molecular Medicine, Norwegian University of Science and Technology, Trondheim, Norway; University of Calgary, CANADA

## Abstract

**Aim:**

Norway has a low prevalence of methicillin-resistant *Staphylococcus aureus* (MRSA) and reporting of all MRSA cases has been mandatory, including infections and carriage, since 1995 and 2005 accordingly. This provides a unique window to study the spread of MRSA in Norway over time. The aim of this study was to analyze the nationwide trends in the molecular epidemiology of MRSA in Norway over a period of 10 years.

**Methods:**

Clinical and epidemiological data as well as bacterial genotype (*spa*-type and PVL) were analyzed for all reported MRSA cases in Norway in the period 2008–2017.

**Results:**

During the study period, there were 15,200 MRSA cases reported in Norway, from 14,386 patients. The notification rate per 100,000 population increased by 15% annually, rising from 14.2 in 2007 to 48.6 in 2017. This increase was primarily driven by MRSA carriage and community-associated MRSA cases. The incidence of invasive infections remained stable and low, at less than 0.5. The incidence of healthcare-associated MRSA showed an increasing trend, while the number of outbreak-related cases, particularly those associated with nursing homes, decreased. Overall, there were significantly more MRSA infections in males than females. Interestingly, there was a significantly higher prevalence of MRSA infections in female young adolescents compared to males. *spa*-typing revealed a very heterogeneous MRSA population (D = 0.97), predominantly impacted by international travel and migration patterns, and less by domestic spread in the community.

**Conclusions:**

This study highlights that Norway, while still classified as a low-prevalence country, has experienced a significant increase in the incidence of MRSA between 2008 and 2017, which can predominantly be attributed to CA-MRSA and MRSA carriage.

## 1 Introduction

*Staphylococcus aureus* is part of the normal bacterial flora in 20–30% of the population, where carriage is typically found in the nose, throat or skin [1]. *S*. *aureus* is also a major human pathogen causing nosocomial and community-acquired infections, ranging in severity from skin and soft tissue infections to bone- and joint infections, pneumonia, endocarditis and bloodstream infections [2].

*S*. *aureus* has a wide array of virulence factors, and the ability to acquire or develop resistance to all classes of clinically relevant antibiotics [3]. Presence of the staphylococcal cassette chromosome *mec* (SCC*mec*), a genomic island that holds *mecA*/*mecC*-genes encoding methicillin resistance, is the defining trait of methicillin-resistant *S*. *aureus* (MRSA) [4, 5]. MRSA are resistant to all beta-lactam antibiotics, including penicillins and cephalosporins, which can lead to delayed, limited options for or failure of treatment [3]. Infections due to antibiotic-resistant bacteria such as MRSA are thus associated with increased morbidity and mortality in healthcare institutions, and the spread of these pathogens is considered a major threat to modern healthcare [6].

Norway has a very low prevalence of MRSA compared to other countries [7], and a “search and destroy” policy in order to prevent MRSA from becoming endemic in healthcare institutions. This involves risk-based screening, contract tracing, isolation of MRSA positive patients and work-restrictions and decolonization treatment of MRSA positive healthcare personnel [8]. Furthermore, all new laboratory confirmed cases of MRSA, including both infections and carriage, are reported to the Norwegian Surveillance System for Communicable Diseases (MSIS), and all MRSA strains are confirmed and genotyped at the Norwegian MRSA reference laboratory. This provides a unique window into the spread of MRSA in Norway over time, and the aim of the current study was thus to analyze trends in the molecular epidemiology of MRSA in Norway based on 10 years of surveillance. Specific objectives included describing temporal changes in genotypes during the study period, and detecting potential associations between MRSA genotype and sex, age groups, infection or carriage, country of acquisition and outbreaks in healthcare institutions.

## 2 Materials and methods

### 2.1 Study design and population

In this study, a case was defined as laboratory-confirmed MRSA notified to MSIS and/or MRSA strains confirmed by the Norwegian MRSA reference laboratory. All cases of MRSA in Norway from 2008 to 2017 were included, limited to the first case per year per individual.

### 2.2 Clinical and epidemiological data

Clinical and epidemiological data on all cases was collected from MSIS (accessed 29.10.2021) and request forms from the referring laboratory or treating physician (accessed through the laboratory information system, LIMS, 22.11.2021). The Information from MSIS included age, sex, admission to hospital or nursing home, place of acquisition and if part of a known outbreak (from the Norwegian outbreak rapid alert system Vesuv) [9]. The data obtained from the LIMS included sample date, sampling site/type of sample and laboratory results. The study group had access to identifiable information after data collection for the purpose of merging the two data sets correctly. All MRSA cases were categorized as carriage, infection, invasive infection or unknown based on sampling site/type of sample. Age groups were based on categories defined by Diaz *et al*. [10]. Due to lack of temporal data for hospitalized patients and nursing home stays, a broad definition of healthcare-associated MRSA (HA-MRSA) was used. Accordingly, a case was categorized as HA-MRSA if the diagnosis occurred during a hospital or nursing home stay, or if MRSA was detected in healthcare workers (HCWs). Conversely, community-acquired MRSA (CA-MRSA) encompassed all other cases not falling within the HA-MRSA classification.

### 2.3 Bacterial strains, PCR and *spa*-typing

Bacterial strains were cultured on blood agar at 35°C, after which extraction of DNA was performed by heat lysis. Briefly, a few colonies were suspended in molecular grade water and heated to 95°C for 15 minutes with shaking (300 rpm). After centrifugation at 14,500 rpm for 2 minutes, the supernatant was collected. Confirmation of MRSA was performed with a multiplex conventional PCR detecting the *mecA* gene, the *S*. *aureus*-specific *spa* gene, and the Panton-Valentine leukocidin (PVL) genes lukSF-PV, followed by gel electrophoresis [11]. For strains that were *mecA* negative, *mecC* PCR [5] was additionally performed.

All strains were *spa*-typed according to Harmsen et al. [11] with primers *spa*-1113f and *spa*-1514r [12] using the Ridom StaphType software and SpaServer [13]. The *spa*-types were assigned to known sequence types (ST) and/or clonal complexes (CC) based on Ridom Staphtyper [12] and pubMLST [14, 15] databases. If a *spa*-type could not be assigned to a ST or CC, multi locus sequence typing (MLST) was performed, and CC was assigned using eBURST [16] software. A minimum spanning tree (MST) based on *spa*-repeats was constructed using Based Upon Repeat Pattern (BURP) clustering [17] with the Ridom SeqSphere + software [18]. To calculate genotypic diversity, Simpson’s diversity index [19] was used.

### 2.4 Statistical analyses

Fisher’s exact test was used for testing associations between the most frequent *spa*-types (≥50 cases) and epidemiological variables (infection, carriage, HA, CA, outbreak, sex and age < 1). Binary logistic regression was used for testing interactions between age and sex, and the Chi-Square test (crosstable analysis) was used for testing for sex differences within age groups. Statistical analyses were performed using R studio v1.4 and IBM SPSS Statistics v29.0.0.0. The Benjamini-Hochberg method was used to correct for multiple hypothesis testing, with adjusted p-values < 0.05 regarded as statistically significant.

## 3 Results

In the 10-year study period a total of 15,200 MRSA cases were reported in Norway, from 14,386 patients (Table 1). The majority of patients had a single case of MRSA (95%), whereas 4% had two cases, and 0.6% of patients had three or more cases of MRSA. Among patients with multiple cases, 609 (87%) had the same *spa*-type, 84 (12%) had a different *spa*-type within the same CC and only 49 (7%) had a different *spa*-type of a different CC. Most commonly, the detection of multiple MRSA cases in a patient occurred in two consecutive years (61%), while 181 patients (26%) had an interval of 3–4 years between detection and 88 patients (12%) had an interval of ≥ 5 years between detections.

**Table 1:** Clinical and epidemiological information on all MRSA cases in Norway 2008–2017. Number (n) of cases and percentages (%) are given, with decimal values (> 2) rounded to the nearest whole number.

MRSA patients and cases				n	%
**Patients**	Total			14386	100%
** **	Female	Total		7173	50%
** **		Carriage		4187	58%
** **		Infection		2414	34%
** **		NA		717	10%
** **	Male	Total		7211	50%
** **		Carriage		3593	50%
** **		Infection		2993	42%
** **		NA		799	11%
** **	Sex unknown	NA		2	0.0%
** **	Number of cases per patient	Single MRSA case		13689	95%
** **		Two MRSA cases		605	4%
** **		Three or more MRSA cases		92	0.6%
** **		Multiple MRSA cases per patient		697	5%
**Cases**	Total			15200	100%
** **	Reported to MSIS			14992	99%
** **	Strain to MRSA reference lab			14810	97%
** **	Methicillin resistance gene	*mecA*		14470	95%
** **		*mecC*		16	0.1%
** **		NA		714	5%
** **	PVL positive	Carriage		1937	13%
** **		Infection		3226	21%
** **	PVL negative	Carriage		6218	41%
** **		Infection		2265	15%
** **	PVL	NA		1554	10%
** **	Healthcare associated	Total		4566	30%
** **		Admitted to hospital		3004	20%
** **		Healthcare personnel		933	6%
** **		Nursing home patient		629	4%
** **	Community associated	Total		10634	70%
** **	Outbreak-associated			299	2%
**Sampling sites**	Carriage	Total		8162	54%
** **			Nose	3576	44%
** **			Throat	3792	47%
** **			Perineum	779	10%
** **			Combination	15	0.2%
** **	Infection	Total		5498	36%
** **			Wound	3189	58%
** **			Abscess	1290	24%
** **			Puss	335	6%
** **			Eye	201	4%
** **			Other non-invasive	361	7%
** **			Blood culture	93	1.7%
** **			Other invasive	29	0.5%
** **	Infection/carriage	NA		1540	10%
**Place of acquisition**	Norway			4199	28%
** **	Abroad			3878	26%
** **		Europe (other than Norway)		896	6%
** **		Africa		430	3%
** **		Asia		1872	12%
** **		North-America		119	0.8%
** **		South and Central America		183	1%
** **		Oceania		26	0.2%
** **		Country not specified		352	2%
** **	NA			7123	47%

**Fig 1:**
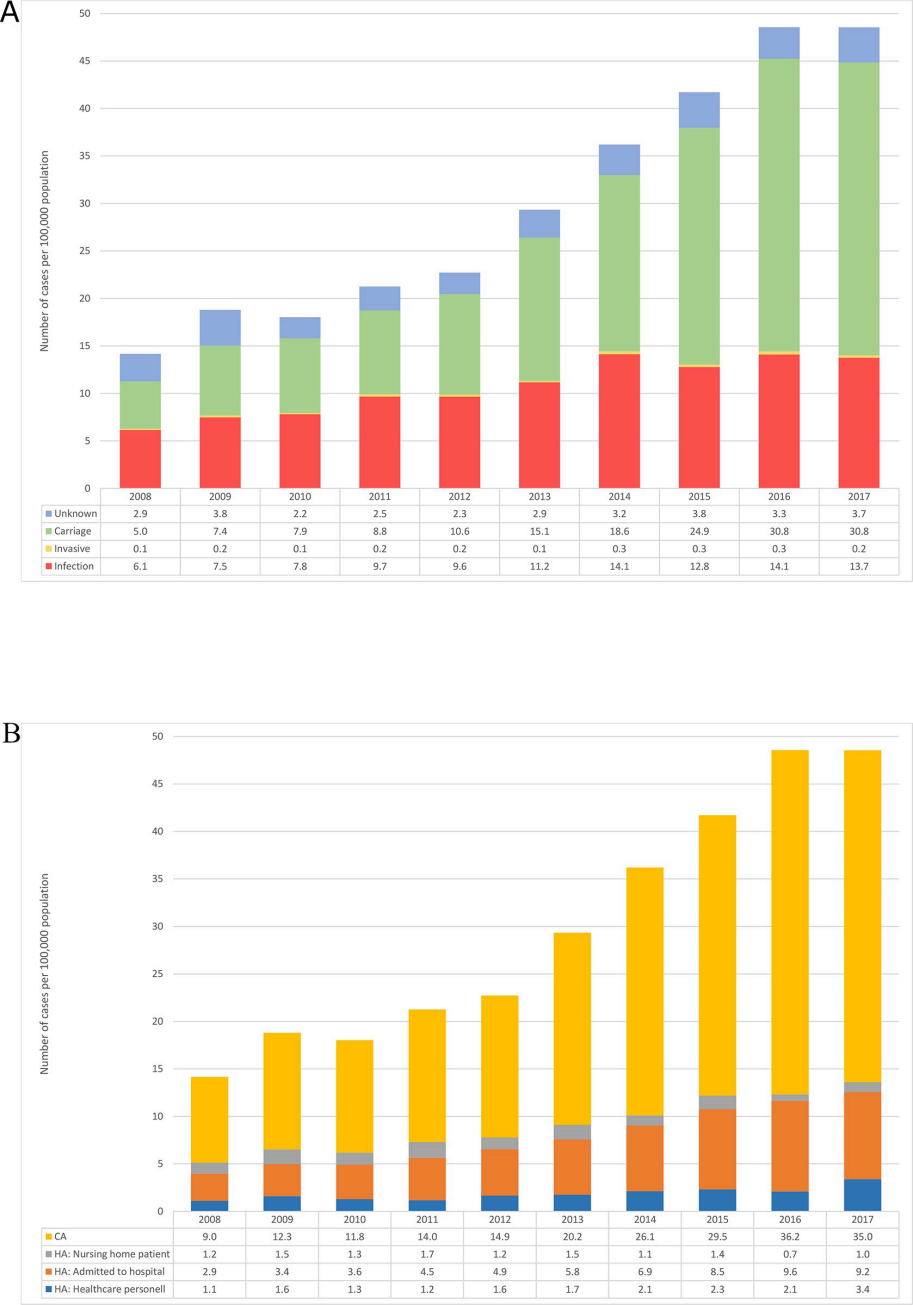
Number of MRSA cases per 100,000 population per year in Norway in 2008–2017. MRSA cases categorized as infection, invasive infection or carriage (a) and as healthcare-associated (HA) or community associated (CA) MRSA (b).

From 2008 to 2017, an increasing trend in notification rates of MRSA was observed, with an annual increase of 15% per 100,000 population. The notification rate rose from 14.2 in 2008 to 48.6 in 2017, resulting in an overall increase of 243% (Fig 1A). The primary driver of this increase was MRSA carriage, which accounted for 75% of the overall rise. In contrast, the rate of infections contributed to 22% of the overall increase. Notably, the incidence of invasive infections remained stable throughout the observed period, consistently below 0.5 per 100,000 population.

Sampling sites for MRSA carriage were mainly nose (44%) and throat (47%), while perineum was less common (10%) (Table 1). For MRSA infections, the most common sampling sites were wound (58%) and abscess (24%). Puss (6%) and eye (4%) were also relatively common categories, while blood culture (1.7%) and other invasive infections (0.5%) were less common.

Of the MRSA cases that were confirmed with *mecA*/*mecC* PCR, the majority were positive for the *mecA* gene (99.9%), while only 16 cases (0–3 per year) were positive for the *mecC* gene (0.1%) (Table 2). For the remaining cases, there was no strain available for *mecA*/*mecC* PCR (5%). The proportion of MRSA cases that were positive for the toxin and epidemiological marker PVL ranged from 45–32%, showing a decreasing trend since a peak in 2011 (Table 2). The PVL positive proportion was significantly higher (p < 0.00001) for MRSA infections (59%) than for MRSA carriage (24%).

**Table 2:** Temporal trends in molecular epidemiology of MRSA cases in Norway in 2008–2017. The table shows frequency of *mecA*, *mecC*, PVL (infection and carriage) for all MRSA cases confirmed with PCR (n = 14,486). Decimal values (> 2) were rounded to the nearest whole number.

		2008	2009	2010	2011	2012	2013	2014	2015	2016	2017	Total
***mecA***		99.80%	99.60%	100.00%	99.80%	99.80%	99.90%	99.90%	99.90%	99.90%	100.00%	99.90%
***mecC***		0.20%	0.40%	0.00%	0.20%	0.20%	0.10%	0.10%	0.10%	0.10%	0.00%	0.10%
**PVL positive**	**Total**	40%	38%	38%	45%	44%	39%	39%	36%	32%	32%	37%
	**Infection**	31%	28%	26%	32%	29%	25%	26%	20%	19%	19%	24%
	**Carrier**	10%	10%	12%	13%	15%	14%	13%	15%	13%	13%	13%
**Most frequent *spa*-types**	**t002**	11%	11%	10%	11%	11%	9%	9%	10%	9%	7%	9%
	**t019**	10%	6%	9%	11%	11%	10%	8%	7%	4%	4%	7%
	**t008**	10%	11%	8%	9%	6%	6%	7%	6%	5%	5%	7%
	**t223**	4%	3%	4%	4%	4%	5%	5%	8%	9%	9%	6%
	**t127**	2%	2%	4%	2%	4%	4%	6%	5%	6%	5%	5%
	**t304**	4%	2%	2%	2%	2%	2%	3%	4%	8%	6%	4%
	**t044**	7%	5%	6%	6%	3%	2%	4%	4%	3%	3%	4%
	**t437**	3%	3%	2%	3%	3%	3%	3%	2%	3%	3%	3%

**Fig 2:**
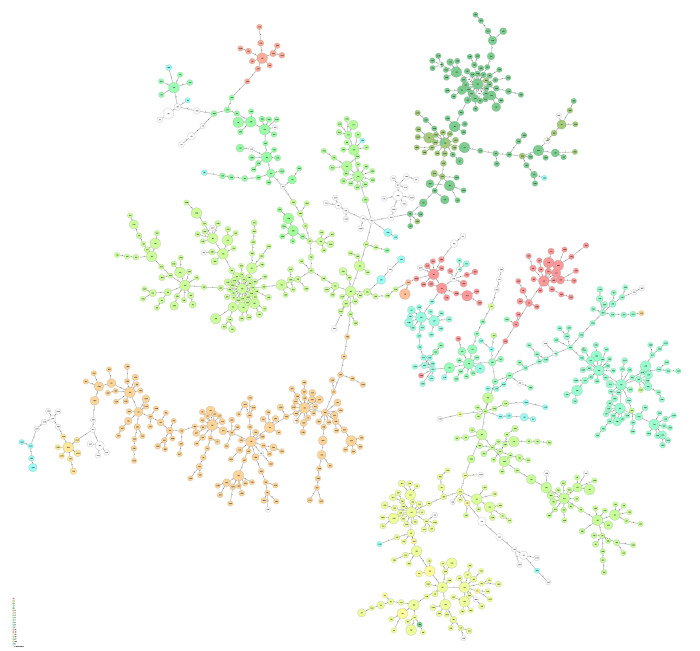
Minimum spanning tree with log distances based on BURP alignments of *spa*-repeats of MRSA cases in Norway 2008–2017. Only *spa*-types with more than five repeats were included (n = 14,140). Nodes are sized according to number of cases, and coloured according to assigned clonal complex as shown in the legend.

Results from *spa*-typing of all MRSA strains revealed considerable heterogeneity (D = 0.97), with 1051 different *spa*-types and four *spa* non-typeable cases (Table 3, Fig 2). Most *spa*-types (90%) were found on average less than 10 times per year. The most frequent *spa*-types overall were t002 (9%), t019 (7%), t008 (7%), t223 (6%), t127 (5%), t304 (4%), t044 (4%) and t437 (3%), which together accounted for 40–50% of the total per year (Table 2). In line with this, the major clonal complexes were CC5 (18%), CC22 (13%), CC5 (18%), CC30 (12%), CC1 (9%), and CC45 (5%). While the *spa*-types t127, t223 and t304 increased in relative frequency throughout the study period, a declining trend was observed for *spa*-types t002, t008 and t019. There were 191 MRSA samples with *spa*-types assigned to CC398 (1.3%), of which 66% were PVL negative and 34% were PVL positive, including mainly t034 (41%) and t011 (18%) (Table 3). There were 96 samples (0.6%) with *spa*-types assigned to *S*. *argenteus* clonal complexes, including CC2250, CC2793, CC1223, and CC1594 (Table 3).

**Table 3:** *spa*-types and associations to clinical and epidemiological data for MRSA cases in Norway in 2008–2017. Number (n) of cases and percentages (%) are given, with decimal values (> 2) rounded to the nearest whole number. Median ages are shown for each *spa*-type within a clinical or epidemiological group. P-values reflect the statistical association (Fisher’s exact test) of a clinical or epidemiological variable (first column) to a *spa*-type, with Benjamini-Hochberg-corrected p-values < 0.05 regarded as significant.

*Spa*-type		Clonal complex	Median age	n	%	P-value
**Total**			31	15200	100%	-
**Unique**			-	1051	7%	-
**Most frequent**	t002	CC5	35	1397	9%	-
** **	t019	CC30	32	1078	7%	-
** **	t008	CC8	34	999	7%	-
** **	t223	CC22	26	937	6%	-
** **	t127	CC1	32	699	5%	-
** **	t304	CC6	24	436	3%	-
** **	t304	CC8	38	206	1.4%	-
** **	t044	CC80	28	582	4%	-
** **	t437	CC59	30	421	3%	-
** **	NA		28	422	3%	-
** **	Multiple	CC398	35	191	1.3%	-
** **	Multiple	CC2250/2793/1223/1594	33	95	0.6%	-
**Associated to infection**	t002	CC5	35	524	41%	1.75E-38
** **	t105	CC5	32	54	42%	1.05E-02
** **	t852	CC22	36	43	44%	2.73E-06
** **	t148	CC72	56	27	47%	2.16E-02
** **	t024	CC8	38	42	48%	1.76E-04
** **	t021	CC30	21	84	49%	1.50E-19
** **	t044	CC80	31	297	53%	1.94E-11
** **	t121	CC8	30	42	56%	3.79E-07
** **	t311	CC5	30	38	59%	2.86E-02
** **	t008	CC8	34	578	62%	1.72E-02
** **	t437	CC59	32	253	65%	2.40E-03
** **	t657	CC1	32	136	66%	1.98E-07
**Associated to carriage**	t304	CC6/CC8	27	506	82%	6.09E-40
** **	t386	CC1	26	77	78%	7.28E-59
** **	t701	CC6	31	45	75%	4.30E-04
** **	t026	CC45	35	118	73%	6.93E-08
** **	t067	CC5	69	71	69%	2.47E-02
** **	t127	CC1	28	443	69%	1.68E-07
** **	t032	CC22	65	100	66%	5.05E-04
** **	t1339	CC88	25	64	65%	8.65E-03
** **	t015	CC45	30	58	60%	2.06E-50
** **	t034	CC398	39	77	60%	5.15E-03
**Associated to HA**	t127	CC1	35	234	33%	4.24E-02
** **	t002	CC5	57	483	35%	4.73E-05
** **	t442	CC5	70	33	48%	4.82E-03
** **	t003	CC5	46	27	54%	1.11E-03
** **	t148	CC72	62	33	54%	3.12E-04
** **	t067	CC5	75	72	57%	2.70E-09
** **	t032	CC22	71	113	60%	1.14E-16
** **	t2958	CC5	84	37	66%	1.89E-07
** **	t065	CC45	82	40	76%	1.08E-10
**Associated to CA**	t034	CC398	32	121	86%	2.21E-04
** **	t186	CC88	13	55	85%	3.73E-02
** **	t1339	CC88	25	84	84%	9.35E-03
** **	t852	CC22	28	85	83%	1.37E-02
** **	t044	CC80	27	469	81%	1.97E-07
** **	t437	CC59	29	322	76%	2.21E-02
** **	t223	CC22	23	713	76%	4.78E-04
** **	t019	CC30	30	816	76%	4.78E-04
Associated to outbreak*	t304	CC6/CC8	80	38	6%	4.98E-09
** **	t034	CC398	40	28	20%	2.25E-19
** **	t528	CC59	77	21	84%	2.33E-31
** **	t032	CC22	46	20	11%	4.98E-09
** **	t065	CC45	82	13	25%	1.67E-10
** **	t613	CC22	74	10	91%	8.85E-16
** **	t688	CC5	28	10	5%	1.58E-02
**Associated to male sex**	t019	CC30	36	601	56%	1.10E-02
Associated to age < 1*	t127	CC1	0	51	7%	9.45E-06
** **	t688	CC5	0	16	8%	1.88E-02

**Fig 3:**
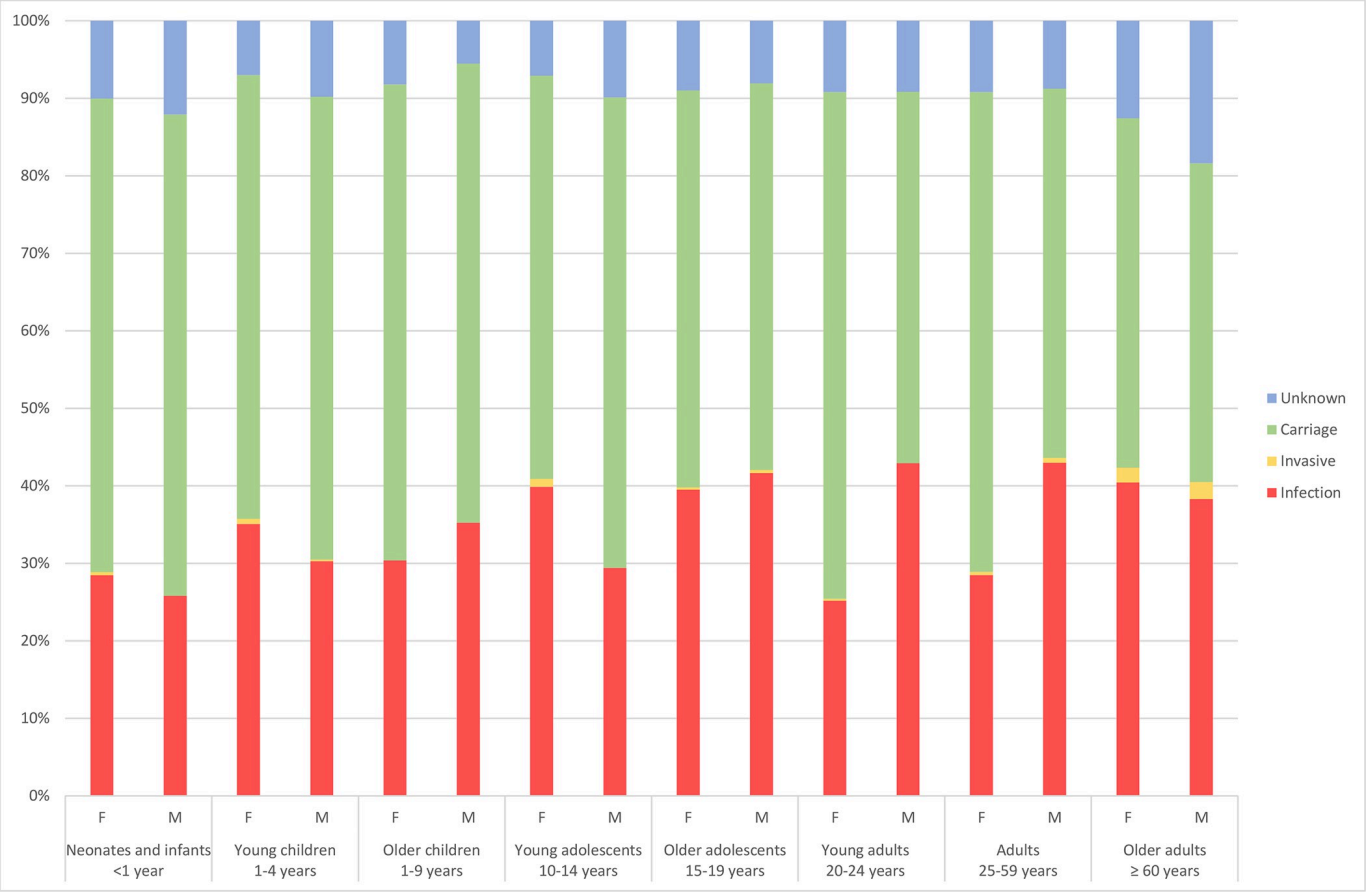
Distribution of MRSA infection and -carriage between sexes within defined age groups. All MRSA cases in Norway 2008–2017, male (M) or female (F), were categorized into age groups based on Diaz *et al*. [10].

The age of all MRSA cases ranged from 0–109 years, with a median age of 31 years (Table 3). Overall, there was an even proportion of male (50%) and female (50%) cases of MRSA (Table 1). We observed a significantly higher percentage of MRSA infections (including invasive infections) in males than in females (56% vs 45%, p<0.001), however the sex-difference varied significantly between different age groups (Fig 3, p<0.01). The higher proportion of MRSA infections in males was observed among young adults (20–24 years) (47% vs. 28%, p<0.001) and adults (25–59 years) (Fig 3) (48% vs. 32%, p<0.001). In contrast, females exhibited a significant higher proportion of MRSA infections among young adolescents (10–14 years) (44 vs. 33%, p = 0.004). No statistically significant associations were observed for other age groups.

Of all MRSA cases, 36% were classified as non-invasive infections, 0.7% as invasive infections, 54% as carriage and 10% as unknown (Table 1). Some of the frequent *spa*-types (i.e. ≥50 cases in total) were associated with infection (Table 3), including *spa*-type t002 (41% infections), t021 (49%), t044 (53%), t121 (56%) and t657 (66%). Conversely, MRSA *spa*-types associated with carriage included t386 (78%), t015 (60%), t304 (82%) and t127 (69%). No *spa*-types were significantly associated with invasive infection, however the major groups of MRSA from invasive infections were t002 (14%), t019 (11%) and t008 (9%), and these were mainly detected from blood culture.

Of all MRSA cases, 30% had an epidemiological link to the healthcare system at the time of sampling, and were thus defined as HA-MRSA (Table 1). Of these, a majority (20%) were admitted to hospital, while 6% were healthcare personnel and 4% were nursing home patients. The 70% of remaining cases were defined as CA-MRSA. Throughout the study period, the incidence of HA-MRSA showed an increasing trend, from 5.2 per 100 000 in 2008 to 13.6 in 2017, of which MRSA in patients admitted to hospital was the major contributor (Fig 1B). CA-MRSA however showed a drastic increase, from 9.0 per 100 000 in 2008 to 35.0 in 2017. Some *spa*-types were statistically overrepresented within healthcare-associated MRSA, including MRSA t032 (60% HA), MRSA t065 (76%) and MRSA t067 (57%) (Table 3). Of these, t032 was found in all HA categories, whereas t067 was detected mainly in hospital admitted and nursing home patients and t065 was found mainly in nursing home patients. Notably, these genotypes were detected in older patient populations (median age ≥ 70 years).

**Fig 4:**
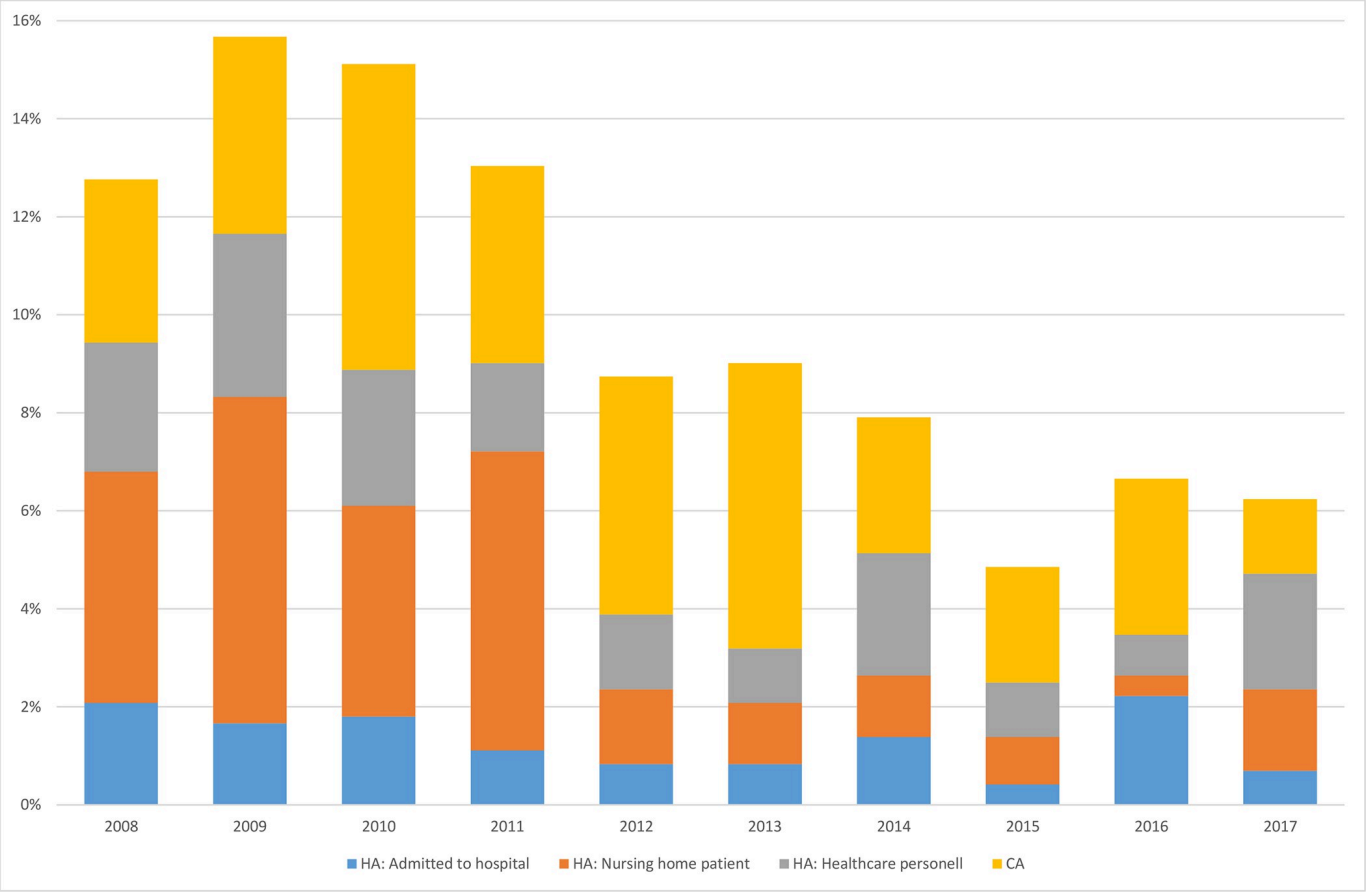
Proportions of outbreak-related MRSA cases per year classified as HA-MRSA or CA-MRSA in Norway 2008–2017.

A total of 299 outbreak-related MRSA cases (2%) belonging to 53 different outbreaks were reported overall (Table 1), with a median outbreak size of only 4 cases (range 1–31 cases). The number of outbreaks and outbreak-related cases decreased markedly from the start of the period (2008–2011) to later in the period (2012–2017), with HA-MRSA and in particular nursing home-related outbreaks contributing to this decline (Fig 4). There was a large heterogeneity of genotypes associated to outbreaks, with a total of 38 different *spa*-types detected. The most frequent *spa*-types which were significantly associated with outbreaks were t304 (6% outbreak-associated), t032 (11%) and t688 (5%) (Table 3). Several of these genotypes were detected in older patient populations (median age ≥ 70 years). Two MRSA *spa*-types were significantly associated with the age group neonates and infants (< 1 year), including t127 and t688 (Table 3).

**Fig 5:**
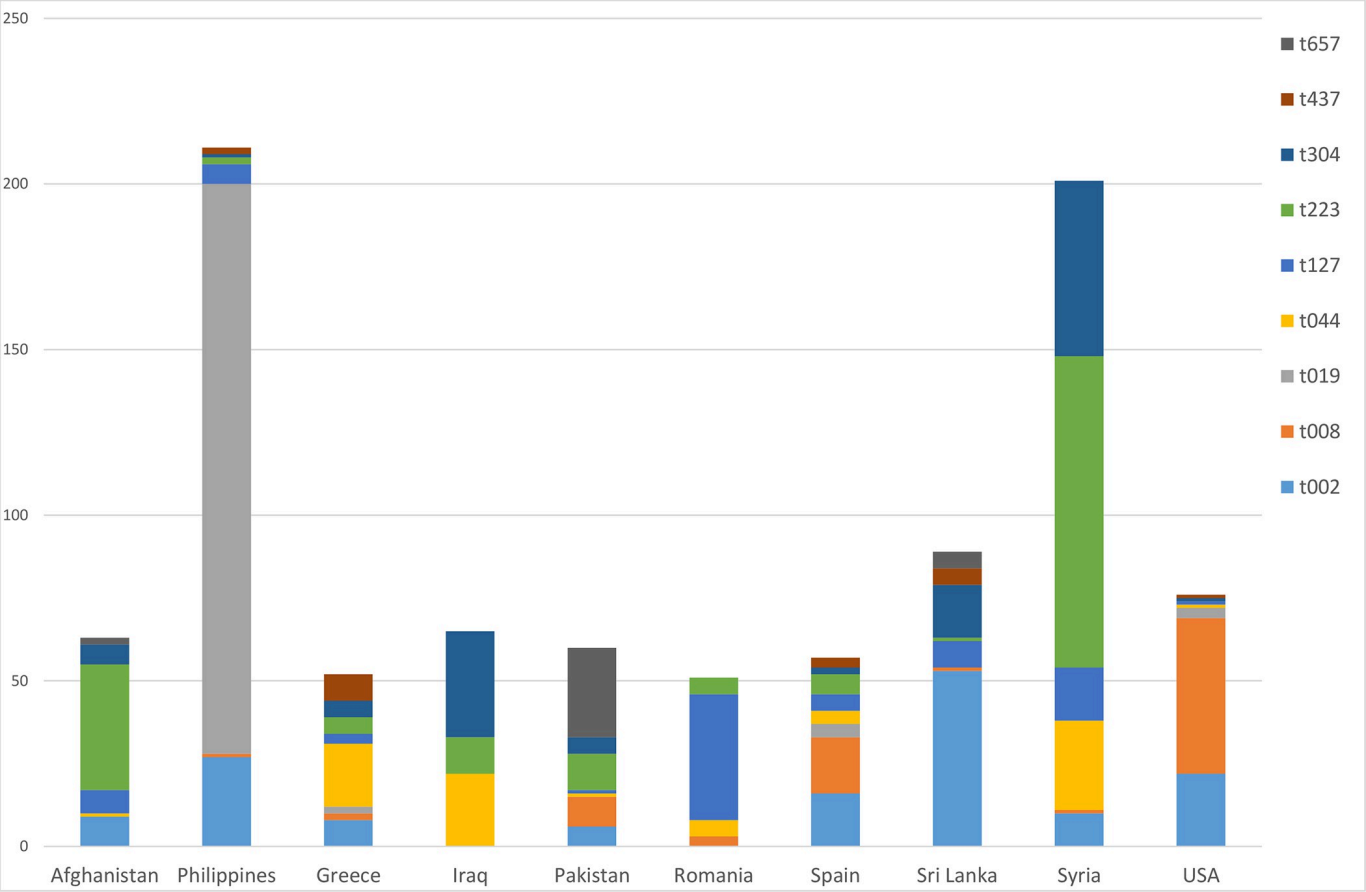
Number and *spa*-type of MRSA cases acquired abroad. Only spa-types with ≥50 cases and countries with ≥50 cases were included.

Of all MRSA cases in the study period, 4199 (28%) were registered as acquired in Norway and 3878 (26%) as acquired abroad, while the remaining cases (47%) had no information on place of acquisition (Table 1). Of the cases that were acquired abroad, Asia (48%) and Europe (23%) accounted for the largest groups, with the Philippines (10%), Syria (9%) and Spain (6%) being the main countries (Fig 5). The *spa*-types which were significantly associated to a specific country included MRSA t019 from the Philippines (46% associated with Philippines), t127 from Romania (54%), t002 from Sri Lanka (50%), t223 and t304 from Syria (28 and 16% accordingly) and t008 from USA (46%) (Table 4).

**Table 4:** *spa*-types and associations to country for MRSA cases in Norway in 2008–2017. Number (n) of cases and percentages (%) are given, with decimal values (> 2) rounded to the nearest whole number. P-values reflect the statistical association (Fisher’s exact test) of a country (first column) to a spa-type, with Benjamini-Hochberg-corrected p-values < 0.05 regarded as significant.

Association to country*:	*spa*-type	Clonal complex	Median age	n	%	P-value
**Afghanistan**	t223	CC22	16	38	24%	3.45E-12
**Brazil**	t002	CC5	20	16	31%	3.28E-07
	t008	CC8	34	11	22%	3.37E-04
**Cuba**	t008	CC8	41	25	58%	2.17E-19
**Egypt**	t223	CC22	29	17	22%	2.76E-05
**Philippines**	t019	CC30	32	172	46%	1.75E-119
**Greece**	t044	CC80	36	19	26%	8.09E-12
**India**	t657	CC1	30	24	21%	1.57E-19
**Iraq**	t304	CC6/CC8	28	32	20%	8.46E-13
	t044	CC80	26	22	14%	3.54E-08
**Macedonia**	t002	CC5	22	11	38%	3.16E-06
**Pakistan**	t657	CC1	36	27	23%	1.35E-23
**Poland**	t437	CC59	32	16	21%	1.13E-10
**Romania**	t127	CC1	30	38	54%	9.91E-37
**Russia**	t223	CC22	24	30	67%	2.18E-24
**Somalia**	t044	CC80	28	13	23%	8.28E-08
**Sri Lanka**	t002	CC5	26	53	50%	2.82E-33
	t304	CC6/CC8	23	16	15%	4.20E-05
**Syria**	t223	CC22	23	94	28%	5.49E-36
	t002	CC5	25	10	3%	2.07E-03
	t304	CC6/CC8	22	53	16%	3.98E-16
	t044	CC80	24	27	8%	5.47E-05
**Thailand**	t019	CC30	38	14	12%	4.37E-02
	t008	CC8	47	15	13%	7.43E-03
	t437	CC59	24	10	9%	9.49E-04
**Turkey**	t008	CC8	49	10	15%	8.92E-03
**USA**	t002	CC5	72	22	21%	2.82E-06
	t008	CC8	31	47	46%	7.89E-30
**Vietnam**	t437	CC59	33	23	25%	4.36E-17

## 4 Discussion

Norway is considered a low-prevalence country in terms of MRSA incidence. However, we observed a substantial increase in the MRSA incidence from 2008 to 2017. This is in line with what has been reported for several of the Nordic countries [7]. Although this likely reflects a real increase in MRSA cases in Norway, variation in screening practices has possibly also contributed. The majority of the increase in MRSA in the study period was attributed to community-associated cases and carriage, indicating that transmission is mainly occurring in the community. The incidence of MRSA infections was however also increasing, which may be a consequence of the substantial increase in carriage. Notably, the incidence of invasive infections remained relatively constant, below 0.5 per 100,000 population.

The incidence of HA-MRSA increased in the study period, primarily among patients admitted to hospitals. Due to lack of information on admission time, we used a broad definition of HA-MRSA, which might lead to overestimation. Furthermore, in line with the major increase of CA-MRSA in the same period, it is likely that part of the increase in HA-MRSA reflects spillover from the community. A declining trend was however observed for outbreak-related cases, especially in nursing homes. The number of cases related to outbreaks are generally underestimated, especially for small and CA-associated outbreaks, which are less likely to be suspected and thus reported to the Norwegian rapid alert system. Nevertheless, it is possible that increased compliance with infection control measures in health-care facilities and Norway’s “search and destroy” policy are contributing factors to the observed reduction.

Different MRSA *spa*-types were significantly associated to either the healthcare setting or the community setting. In general, the *spa*-types associated with HA-MRSA had a patient population with higher median age (35–84 years) than the *spa*-types associated with CA-MRSA (13–32 years), likely reflecting the differences in setting where these clones were mainly spreading. An example of this is MRSA t032, a well-known HA-MRSA clone common in European countries [13], with a patient population of median age of 71, which was associated both to the healthcare setting and to outbreaks. Of the community associated MRSA *spa*-types, t223 was linked to acquisition from Afghanistan, Egypt, Russia and Syria, and found in a much younger population (median 23 years), likely due to the typically young age of immigrants from these countries.

The MRSA population in Norway was very heterogeneous, as neither of the major *spa*-types detected constituted more than 10% of the total MRSA cases, and most *spa*-types detected (90%) were found less than 10 times per year. These findings indicate that there are few successful clones and limited spread, either in the community or in the healthcare system. A previous study has indeed suggested that spread of MRSA in Norway is self-limiting (reproductive number of 0.68), and that the observed increase in incidence is mainly due to acquisitions from abroad [20]. Based on the data on acquisition of MRSA in this study, 28% of the cases were acquired in Norway, while 26% were acquired abroad. However, this is often registered based on assumption, and for the majority of cases we had no information on place of acquisition. Thus, it is likely that these numbers are underestimated.

There have been major changes in immigration-patterns to Norway during the 10-year-period [21], where the total number of immigrants per year increased from 2008–2011, and decreased from 2011–2017, mainly from Eastern European countries. From 2015–2017 there was a significant increase of immigration from Asia (especially Syria), related to the civil war in Syria. Other countries with large immigrant groups to Norway in the study period include Romania, Somalia and the Philippines. These patterns are to some extent reflected in our findings, where we observe relatively high numbers of *spa*-types associated to specific countries with high immigration to Norway in the period, including MRSA t127 (Romania), t019 (Philippines), t002 (Sri Lanka), t223 and t304 (Syria) and t008 (USA).

Several studies have previously shown that bacterial infections are more frequent in males than in females [22], and this was also reflected in our study. The causes are described to be multifactorial, stemming from genetic, anatomical, immunological, hormonal and behavioural differences between the sexes [23, 24]. Notably, we observed a significantly greater number of infections in males within the young adult (20–24 years) and adult (25–59 years) age groups, while females exhibited a higher prevalence of carriage strains. A possible contributing factor to this is that these age groups coincide with the prime childbearing years of women, potentially leading to more MRSA testing due to increased interaction with the healthcare system. Another important finding was that in the young adolescents group (10–14 years) we observed significantly higher rate of infections in females than in males. To our knowledge, this has not been previously reported, and could be attributed to using age groups stratified by life stage in this study. Although the reasons behind this finding are unclear, contributing factors may include onset of puberty and modulation of sex hormones on the immune system [23, 25].

We observed that several MRSA *spa*-types were significantly associated either with infection (t002, t021, t044, t121 and t657), or with carriage (t386, t015, t304 and t127). Although there may indeed be biological differences in virulence traits that render some MRSA clones more virulent, this is outside of the scope of this work. The MRSA *spa*-types associated with infection did however not appear to be associated with higher age groups, and included both HA- and CA-MRSA. Among them were some of the most common *spa*-types in Norway, the globally disseminated MRSA clones t002 and t008, as well as the multidrug resistant Bengal Bay clone t657 [26] and the European CA-MRSA clone t044 [7]. No *spa*-types were significantly associated with invasive infection. However, in this study only 108 invasive strains were included, and this number was likely too low for sufficient statistical power. Nevertheless, t002, t019 and t008 were the most frequent MRSA *spa*-types causing invasive infections, and were also among the most frequent *spa*-types in Norway. A characteristic of the invasive infection group was that the patient population were mostly older adults (≥60 years). Thus, invasive infection appears to be linked to high age, and likely with increasing comorbidities in this group.

Among the *spa*-types associated with carriage were MRSA t304 (CC6) and t127. Both of these have been considered CA-MRSA clones, and have also previously been linked to Middle Eastern countries [27, 28] and South-Eastern Europe [29], respectively. The patient population of these *spa*-types were generally young, and thus presumably mostly healthy. However, if many become persistent carriers, it could possibly result in more infections in an older population. Furthermore, it is concerning that these MRSA *spa*-types have been reported to cause relatively large neonatal outbreaks, in Norway (unpublished data) and in Denmark [27, 30]. MRSA t127, along with MRSA t688, was significantly associated with neonates and infants (< 1 year) in this study, the latter also to outbreaks, and has previously been found in maternity wards in Sweden [31], Ireland [32] and Kuwait [33].

## 5 Conclusions

From 2008 to 2017, there has been a notable rise in the incidence of MRSA in Norway. This increase can be primarily attributed to CA-MRSA and carriage, while the incidence of infections and invasive infections have remained relatively low. Overall, there were significantly more MRSA infections in males than females. Interestingly, there was a significantly higher prevalence of MRSA infections in female young adolescents compared to males. The incidence of HA-MRSA has also increased, although it is still below 10 per 100,000 population. This suggests that the Norwegian "search and destroy" policy has been effective in preventing MRSA from becoming endemic in Norwegian healthcare institutions. Global patterns of travel, migration and work have however had large impact on the MRSA epidemiology in Norway, highlighting the importance of national and international surveillance in order to monitor temporal trends and spread of successful clones. Increased use of whole genome sequencing as well as more timely access to epidemiological data will hopefully enable an even more detailed view on the transmission of MRSA as well as a more rapid response to potential outbreaks in the future.
